# Automatically Augmenting Lifelog Events Using Pervasively Generated Content from Millions of People

**DOI:** 10.3390/100301423

**Published:** 2010-02-26

**Authors:** Aiden R. Doherty, Alan F. Smeaton

**Affiliations:** Centre for Sensor Web Technologies, Dublin City University, Glasnevin, Dublin 9, Ireland; E-Mail: alan.smeaton@dcu.ie

**Keywords:** lifelogging, event augmentation, SenseCam, Web 2.0

## Abstract

In sensor research we take advantage of additional contextual sensor information to disambiguate potentially erroneous sensor readings or to make better informed decisions on a single sensor’s output. This use of additional information reinforces, validates, semantically enriches, and augments sensed data. Lifelog data is challenging to augment, as it tracks one’s life with many images including the places they go, making it non-trivial to find associated sources of information. We investigate realising the goal of pervasive user-generated content based on sensors, by augmenting passive visual lifelogs with “Web 2.0” content collected by millions of other individuals.

## 1. Introduction

Almost everything we do these days is in some way monitored, sensed, or logged. We’ve come to accept—or maybe we just ignore—this massive surveillance of our lives from a variety of sensors because it brings us benefits. For example, we have a more secure feeling when we know there is CCTV present, we get itemised billing of our phone usage from phone companies, and we get convenience and even loyalty bonuses as a result of some of our regular store purchases.

Lifelogging is the term used to describe recording different aspects of your daily life, in digital form, for your *own* exclusive personal use. It is a form of reverse surveillance, sometimes termed *sous*veillance, referring to us, the subjects, doing the watching, of ourselves. Lifelogging can take many forms, such as the application which runs on your mobile phone to ‘log’ all your phone activities and then present all your phone-based activities in a calendar format. While there are many technologies which can be used to generate lifelogs, perhaps the most important of the key dimensions for accessing lifelogs, is to structure our lives into *events* or happenings, corresponding to the happenings which occur in our lives. Preparing food, eating a meal, cleaning the kitchen, walking to the train, taking a train journey, sitting in an office desk . . . all these correspond to events which make up our lives and these events make up the retrievable units of a lifelog.

**Figure 1.**
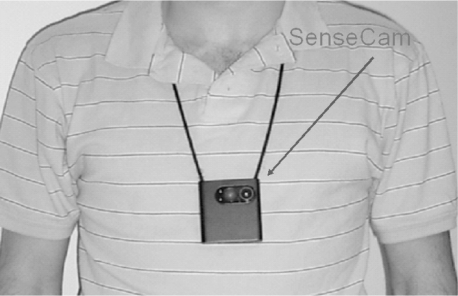
The Microsoft SenseCam.

For over four years we have been working with a small, wearable camera called the SenseCam [1] developed by Microsoft Research in Cambridge, UK that creates a sensor and visual record of the wearer’s day. The SenseCam is worn on the front of the body, suspended from around the neck with a lanyard as displayed in Figure 1. It is light and compact, about one quarter the weight of a mobile phone and less than half its size. It has a camera and a range of other sensors for monitoring the wearer’s environment by detecting movement, temperature, light intensity, and the possible presence of other people in front of the device via body heat. The SenseCam regularly takes pictures of whatever is happening in front of the wearer throughout the day, triggered by appropriate combinations of sensor readings. Images are stored onboard the device, with an average of 2,000 images captured in a typical day, along with associated sensor readings (logged every second).

Given that the SenseCam captures more than 2,000 images and up to 35,000 sensor readings in a typical day, in our work we make this information more accessible by firstly segmenting sequences of images into distinct events/activities e.g., breakfast, meeting, walk in the park, *etc*. [2]. This exploits the fact that the human mind stores memories in the form of events as noted by Zacks, who studies how representation in the brain works *“. . . segmenting ongoing activity into events is important for later memory of those activities . . . ”* [3].

In sensor research we take advantage of contextual information to augment our understanding of given events, and indeed the neuropsychological research community have shown research stating that the human memory operates by associating linked items together [4]. We feel it may be useful to augment an individual’s SenseCam images of an event by linking in images, or videos, from other associated external sources of information in order to further enhance the reviewing experience. For example if a wearer attends a big football match, it may enhance the enjoyment of their recall of the event and provide cues to other memories of the match if they are able to easily view additional photos and videos of the event; similarly if someone is at the Eiffel tower in Paris, their experience of reviewing the event by viewing SenseCam images may be enhanced by photos taken by others in that location, where even a photo of a nearby park bench may be relevant as it evokes memories of the user sitting there. From a clinical viewpoint, such reviewing of past events, recorded using SenseCam and its sensors, and possibly even augmented with images from other sources, has been shown to positively enhance recall in patients suffering from early-stage memory impairment [5].

In a number of fields of sensor research we commonly seek additional sources of associated information to make better informed decisions based on a given sensor’s output. For sensors in the marine environment, we take associated environmental sensor data e.g., tide times, turbidity, wave speed, *etc.*, . . . for sensors on vehicles, we can associate their data with information on traffic at particular junctions to give more contextual information . . . for physiological monitoring of football/tennis matches we combine multiple different types of sensors together (heart rate monitors, video footage of match, sensors to track the player’s movement) to make more informed decisions and to have smarter sensing. However lifelog data is different in that it tracks a user’s life with many images and sensor readings from all the different places they visit and activities they do. Therefore it is very difficult to predict fixed and structured sources of data that may be useful as augmented sources of information. In response to this, to help find associated sources of information we turn to collating images from the internet and particularly websites of user-generated content like Flickr, YouTube, *etc*. So through using simple sensors on the SenseCam, and also mining the small contributions of a large number of individuals to websites with user-generated content, we can enhance the experience of re-viewing past SenseCam events.

To realise the goal of augmenting lifelog events with information from external sources there are a number of individual steps needed. Firstly, we need to know where and when the lifelogged event took place so it is necessary to record location (*via* GPS) and time information along with the captured SenseCam images and its sensor readings. Once we have the location, the user can then be given the option of whether to constrain the augmentation to images at a certain time or to allow images taken at any time to be used for augmentation. The time associated with images taken of an historical building is not so important, whereas the time when images are taken at the location of a football match will be very important as the user will want images captured by other people at that same match, not of other events in the same stadium. With GPS information of where an event is taking place it is then possible to query an external data source for images only within some distance of the location of where the user was, for the particular event.

In the case of an historical building for example, there may be many close-up pictures taken by other people of their friends at that location. We have investigated, at query time, carrying out a form of image similarity matching between the keyframe image for the SenseCam event, and retrieved images from the external data source. If a retrieved image is sufficiently visually similar to the SenseCam image, then it will be displayed to the user as a potentially useful image to augment the lifelog event. This could result in the user being returned a large number of highly ranked relevant images from external data sources but in this paper we present our techniques for addressing this, along with our experimental validation.

Our work contributes to realising the goal of pervasive user-generated content, as passive visual lifelogs are augmented with “Web 2.0” user-generated content collected by millions of other individuals. We present a system that realises the aim of using visual content and sensor readings passively captured by an individual and then augmenting that with web content collected by other individuals. This article describes the system we developed, how it operates, and how effective it is. In section 2 we will detail related work, before describing how we automatically augment lifelog events in section 3. In section 4 we outline how we setup our experiments and our results are discussed in section 5. Finally in section 6 we present conclusions.

## 2. Related Work

**Figure 2.**
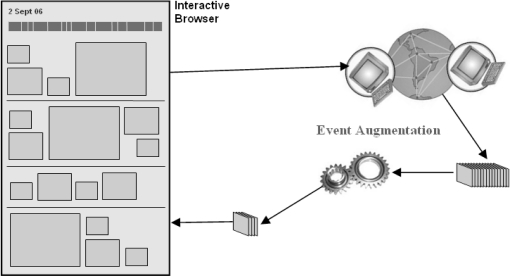
Augmenting lifelog events with images from external sources of information.

The goal of the MyLifeBits project [6] is to store a significant amount of the experiences that an individual will encounter in his or her lifetime in digital format, and then to make that data available for searching, summarising or just retrieval. Therefore the MyLifeBits project is essentially a database that logs many activities such as: Internet browser logging, interface activities, media files played, e-mails sent/received, SenseCam images, GPS location data, *etc*. In MyLifeBits it is possible to augment SenseCam images with other pieces of information such as the location a picture was taken, whether the user was working with a particular application on their PC at the time a picture was taken, *etc*. It is our intention to augment SenseCam images with images from *external* publicly-available data sources (as illustrated in Figure 2), and to investigate whether this is useful to users or not.

Lim *et al.* attempt to recognise images taken by tourists on mobile phones, and then augment the tourist’s photo with a text description of a recognised monument [7]. In this *Snap2Tell* system, they have the STOIC database which contains 5,278 images of 101 Singaporean tourist locations (with a minimum of 16 images per scene). They then do a novel form of matching image patches around salient regions of the images, and return the database image closest to the picture taken by the tourist on their mobile phone. The image is returned along with a text description of the attraction to the phone. O’Hare *et al.* have done something similar [8] where they selected 6 example query images and attempted to identify similar photos from other users’ collections. They filtered firstly based on location, investigating the optimal physical distance to filter results by. There didn’t appear to be a significant difference in filtering by 200 m (smallest distance) or by within the same city (around 5–10 km), possibly somewhat due to the relatively small dataset. They performed an image: image similarity matching between the query image and the resultant images and their main conclusion was that it is necessary to firstly filter by location before attempting matching images based on low-level features. Nini & Batouche have done similar work in that they try to identify (fixed) objects and then provide extra augmented information on those objects [9]. Like the work of Lim *et al.* [7] and of O’Hare & Smeaton [10], a limitation of all these systems is that the datasets of images are of a fixed size. Kawamura *et al.* also produce a memory augmentation paper, but it is based on using RFID tags to identify objects in a *fixed* scene [11].

We expect lifelogging devices to become more commonplace in future, especially since companies like Vicon OMG have announced easy availability of these in the form of the ViconRevue (http://www.omg3d.com/html/IPLicenseagreement.html). Given the phenomenal growth of multimedia content on websites, we introduce the idea of automatically augmenting lifelog events with “Web 2.0” content. We investigate aggregating these small contributions over an enormous scale of users, which can thus automatically enrich the experience of individuals reviewing their trips or other lifelog events, by providing relevant items of information mined from web sources from millions of other individuals.

Kennedy & Naaman detail a clustering method to select images of a location that are both representative and also distinct, by showing images from Flickr taken from multiple view points of places like Big Ben and other landmarks in London [12]. They focus on diversity of results, and in effect the information task is displaying varied images on a browser. The information need in our event augmentation task is different as it is very specific, where users only want to see images of say the Eiffel Tower, and not other regions in Paris.

**Figure 3.**
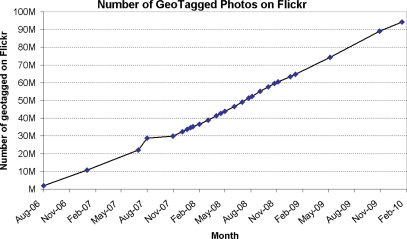
Number of geotagged photos on Flickr.

We attempt to find similar pictures in the same location as those lifelog images taken by a wearable camera using the Flickr (www.flickr.com) and Panoramio (www.panoramio.com) websites. As is reflective of many “Web 2.0” sites, the volume of users and photos on Flickr is astounding. Van Zwol reports that Flickr has 8.5 million registered users, and at peak times there are up to 12,000 images served per second; the record number of photos uploaded per day is more than 2 millions [13]. Flickr also provide users with the opportunity to include the location in the upload of photographs or to drag them onto a map and thus automatically append GPS co-ordinates. There are now over 95 million ‘geotagged’ images on Flickr (at time of writing) and the growth rate is approximately 500,000 new geotagged photos uploaded per week as illustrated in Figure 3. Users can also tag their uploaded images, and given the significant user base on Flickr this can very quickly create a vast collection of tags which are potentially useful in retrieval.

However Flickr is not the only source of user-generated geotagged images. Google Earth also allows users to upload images, which are then accessible on the Panoramio website. *All* of these images are geotagged. Another useful web site is YouTube (www.youtube.com), where millions of users upload *their* own videos. In recognising the shift towards user generated content on the web, Gill *et al.* report that there are 100 millions video views per day on YouTube (which is 60% of the total watched videos on the Internet), and 65,000 new user-tagged videos are uploaded per day [14]. Indeed recent data indicates that 20 hours worth of video content is uploaded every minute to YouTube (www.youtube.com/t/fact_sheet)!

While a very large number of images are now uploaded on the web by many users, the more traditional sources still publish images which are searchable by the major search engines. These index billions of images (e.g. in a clustering paper Liu, Rosenbery, & Rowley of Google used a set of 1.5 billion images from their image index [15]). These indices can now be queried by using publicly available API’s, as can the “Web 2.0” image/video sites [16].

Utilising external sources of information such as the above has the potential to overcome the limitations of previous systems which have been able to augment lifelog images only with limited datasets of information [7]. The significant research challenge addressed in this article is to automatically identify only the images from the Flickr website relevant to a given lifelog event. Afterwards, we also explore augmenting lifelogs with images from other non-geotagged sources on the Internet such as Yahoo!, MSN, and YouTube.

## 3. Augmenting Lifelog Events with Web Images

**Figure 4.**
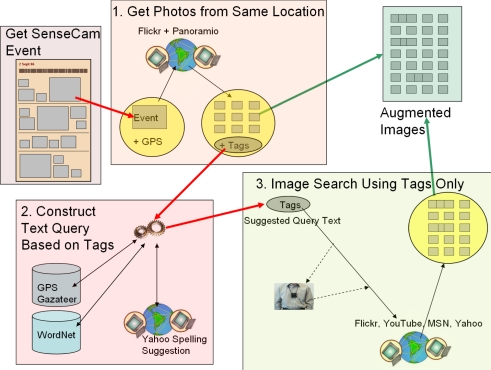
Overview of our Lifelog Event Augmentation Processing.

We now discuss the steps taken when augmenting a lifelog event with images/videos from the Internet. However before detailing how we realise this (summarised in Figure 4) we will first briefly walk through a high level overview of a sample event that a user wanted to augment, so as to enrich his experience when reviewing his trip to Singapore.

**Figure 5.**
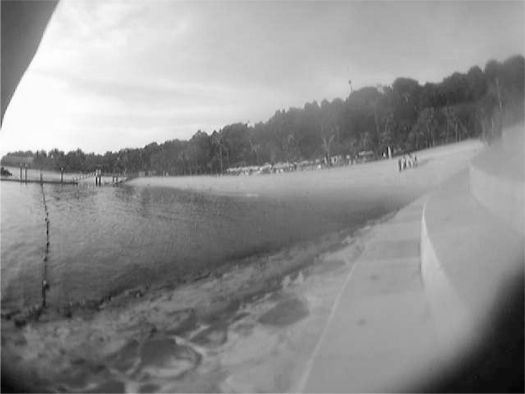
Sample SenseCam lifelog image from event at Sentosa Island, Singapore.

**Figure 6.**
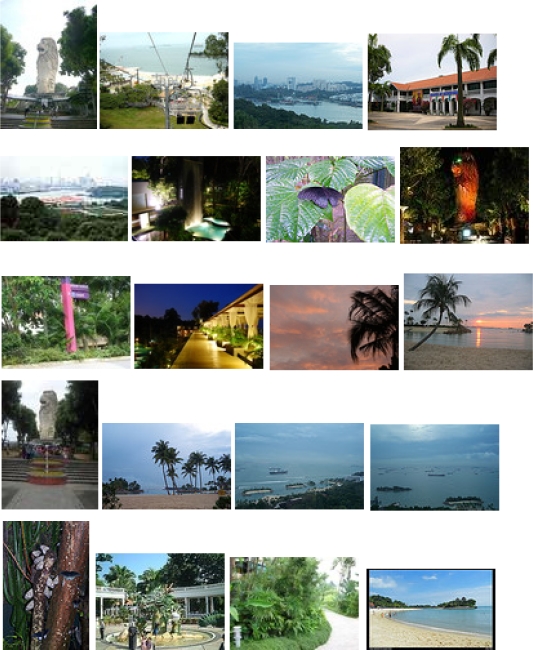
Sample geotagged images of Sentosa Island, Singapore on Panoramio and Flickr.

- **Step 1: Get Photos from Same Location** Consider a real life event where a SenseCam user was on a visit to Sentosa Island in Singapore (see sample image in Figure 5). To augment this event we firstly retrieve a sample of up to 100 images taken in the same location which were made available on the Flickr and Panoramio websites (see 20 random images sampled in Figure 6). In this instance we take 50 sample images from each geotagged source.
- **Step 2: Construct Text Query Based on Tags** Knowing the GPS co-ordinates where the event took place, we can automatically extract the region and country name using a gazetteer [8], which gives us “Pulau Palawan—Singapore (general)—SINGAPORE”. We then make use of WordNet [17] to expand the list of place-name terms to include “Republic of Singapore” and also “Singapore Island”. Knowing the region and country names, which we will use in later in the process, we ignore similar data in the user tags as this does not add any more useful information. Therefore we only consider the tags that are *not* of any variations of the region or country names, and count how many unique individuals used the remaining tags (see Table 1).
  The 4 most commonly occurring tags are selected and also the region name (from the GPS co-ordinates), and in the “Sentosa Island” example we are left with the terms: “Sentosa”, “Island”, “Merlion”, “Beach”, “Singapore”. Finally we spell check each of these terms (using Yahoo! spell checker) so as to allow us retrieve the maximum number of potentially relevant results when performing a text-only search.
- **Step 3: Image Search Using Tags Only** Finally we search the MSN, Flickr, Yahoo!, and YouTube sites with the text query “sentosa island merlion beach Singapore”. For this event 39 of the geotagged images (from Flickr and Panoramio) were relevant, but through the automatically generated text query, we have added another 43 new relevant items (from Flickr, MSN, Yahoo!, and YouTube) to enhance our user’s experience when recalling their visit to Singapore.

Having walked through an example, we will now go through how each of these processing stages (illustrated in Figure 4) are automated.

### 3.1. Download Photos from the Same Location

With the SenseCam device which we use for lifelogging, the time at which images and environmental sensor readings are captured are automatically recorded. With mobile devices now increasingly carrying GPS loggers (e.g., Nokia N95, http://www.nseries.com/products/n95/) it is possible to automatically record the GPS location of our whereabouts on the device itself. Using logs from both of these devices, SenseCam and mobile phone, it is possible to query the Flickr and Panoramio (*i.e.*, Google Earth) websites using location information, through publicly available APIs. This initially returns a set of Flickr and Panoramio photos taken at the same location as the images from the SenseCam event to be augmented. For this work we selected photos within an arbitrary radius of the lifelogged GPS location, which thus offers an obvious focus for future enhancement.

**Figure 7.**
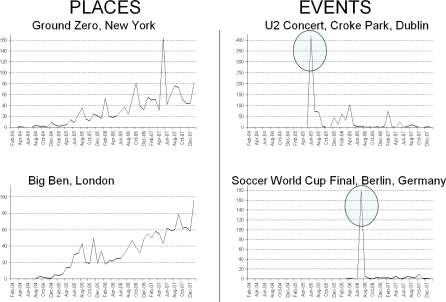
Characteristics of location-driven images *vs*. location+event-driven images.

For some of our lifelogged events, the time of the event may be critical, e.g., attending a sporting event or rock concert, whereas for other events, such as visiting the Statue of Liberty, it is not. On inspection of a sample set of 12 major (time-critical) events, such as sporting events, we noted that 53% of photo uploads of those events occurred within 2 days of the actual event taking place (note some examples of this on the right hand side of Figure 7). After reviewing a number of other time-critical events, we decided to constrain images for such events to be within ±5 days of our lifelog event. In our work we ask users to specify whether an event should be time-constrained or not, however this process can be automated as Rattenbury, Good, & Naaman have developed an approach that can automatically identify if an event is time-critical or not [18].

In selecting the set of query tags to use in the next processing stage it is necessary to initially gather some seed images which will have associated tags. We investigated 3 possible approaches in selecting seed images, namely:

1. **T100:** Considering tags from the top 100 geotagged returned results (as ranked by Flickr and Panoramio (Up to 50 results from Panoramio API (when possible) and the others (50+) from Flickr API))
2. **T25:** Considering the tags from the top 25 results which may be quicker to process
3. **M25:** Using the tags from the top 25 geotagged results that are visually most similar to the lifelog keyframe image (using the set of 100 top-ranked geotagged images returned by Flickr and Panoramio). This requires us to extract image descriptors for 100 Flickr/Panoramio images on the fly, which has a computational overhead. We determine visual similarity between images using the scalable colour MPEG-7 image descriptor [19].

### 3.2. Construct Text Queries Based on Tags

In some circumstances it may be acceptable to return only the Flickr and Panoramio geotagged images to augment an event. However not all images are geo-tagged. In taking a set of 26 sample queries of famous locations (e.g. Eiffel Tower) and events (e.g., FA Cup football final) we discovered that only 22% of the photos of these landmarks and events are geotagged. In fact, given that there were over 2 billion images in total on the Flickr site (http://blog.flickr.net/en/2007/11/) in November 2007, we can infer that only 2% of *all* photos on the entire Flickr database are geotagged. Therefore in wanting to locate as many (relevant) images as possible for event augmentation, we should also search other sources, not just geotagged items.

Since we are initially able to search for geotagged photos taken at the same location as the user’s lifelog event (as described in Section 3.1.), it is then possible to inspect the tags associated with those images, and intelligently construct a new text query. Text annotations are powerful descriptors of image content, and Berendt & Hanser claim that tags are not metadata, but are for all intents and purposes an extension of the content itself [20]. However as Schmitz notes, user-generated tags often contain bad spelling or no spaces between words [21]. Given all the unique tags from each Flickr/Panoramio user, we automatically “clean up” those tags in two steps (While initially it may seem that performing the step of getting initial seed geotagged images (as described in Section 3.1.) may be misguided as only 2% of the collection of Flickr images are geotagged; it should be remembered that this 2% represents an enormous collection of over 95 million images!):

- Firstly, many photo tags in Flickr and Panoramio are of the actual country name or region name, which is not so useful, as we already have GPS information recorded. Also as the majority of tags contain country/region names they create a lot of noise which we want to ignore. Knowing the GPS location where the event took place, we use a gazetteer [8] to automatically extract the associated country and region names e.g., “New York, United States” and then we use WordNet [17] to expand the list of possible tags that users could have used to mark this place, e.g., “United States” expands to: “US”, “USA”, “United States of America”, *etc*.
- Of the remaining tags, the 4 most commonly occurring across different users are then considered as these are likely to be most relevant to a given event query. Many times those tags may be misspelt or contain spaces, therefore we use the Yahoo! spelling suggestion API to correct any erroneous tags e.g., “statue of liberty” becomes “statue of liberty”.

In essence this process is a form of pseudo relevance feedback which is common in information retrieval where the results from an initial query are mined to construct a more elaborate and detailed query. In our case an initial query is formed through searching by location (using GPS), and a new query is then constructed by looking at commonly occurring tags in the initial set of geotagged images. This is a well known technique in the information retrieval field. The tag-selection strategy here may appear quite straightforward (the most popular tags minus the geo terms), and certainly more sophisticated methods should be investigated in future.

### 3.3. Image Search Using Tags Only

Given a set of relevant tags, we can now construct text queries, allowing us to search for images from websites with publicly available API’s such as YouTube, MSN, and Yahoo!, in addition to the images we get from websites with geotagged material like Flickr and Panoramio. This provides 5 potential sources of images, from which we can augment a lifelog event. We allow our users to alter the suggested text query (in certain instances), and then we provide users with a number of candidate images from the 5 different sources to supplement *their* experience of reviewing *their* lifelog events. We now explore how these can be used in practice.

## 4. Experimental Setup

To evaluate the effectiveness of our event augmentation, and also to help answer a number of the research questions we posed, we now describe the setup of experiments where eleven users collected lifelog data over a period of 2 years. Many wore the SenseCam device sporadically (generally at times of interest, e.g., conference trips, holidays, *etc*.), and in total we collected 1,937,770 images which were automatically segmented into 22,911 events (see Table 2). We realise that user 1 may appear to skew the dataset and this will be addressed in more detail while evaluating our final proposed system in Section 5.4. However we include user 1 for two reasons: (1) To illustrate the volumes of data that can be gathered by enthusiastic lifeloggers; and (2) The number of place/time tagged events (described in next paragraph) from user 1 is equivalent to that of the other 10 users and we believe they add to the validity of our conclusions.

**Figure 8.**
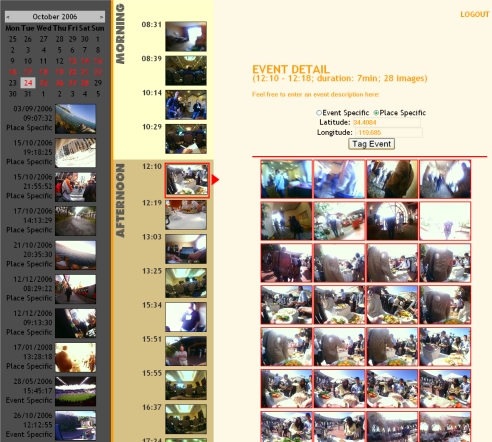
SenseCam browser to mark events for annotation.

Users were each asked to select a number of events that they would like to have automatically augmented with other images from external sources of data. They were presented with an event based browser (illustrated in Figure 8) to sift through their SenseCam images. The calendar allows the user to browse to a day of interest. The vertical column of images in the centre then displays each event for the selected day, and once clicked all images from the event are shown on the right of the screen. Users can select events for augmentation by clicking on the relevant radio button as to whether it is “event specific” or “place specific”, and then by clicking on the “tag event” button. A list of tagged events is displayed under the calendar on the left hand side of the page. In total, 67 events were selected by our 11 users to be augmented (see the “place” and “time” tagged columns in Table 2), with 11 of those events time-specific (e.g., a sporting event, rock concert, *etc*.).

**Figure 9.**
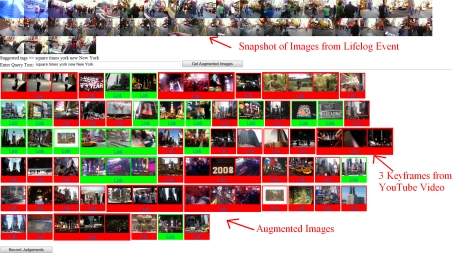
Judging results for event augmentation.

The search for augmented images was then run for each event in our dataset. Two variations of our system were explored, where text from image tags was used directly as the query and where the user had the opportunity to amend the suggested query text. Users were presented with candidate images (10 from each data source), (see Figure 9) in which they had the option to select images relevant to their lifelog event, which were in turn highlighted with a green background. By moving the cursor over a candidate image the user was provided with a text description taken from the webpage that the image belonged to. Below each augmented image there is a “Link” button which opens the original source of that image in a new browser window. 4,963 candidate augmentation images/videos were retrieved for presentation to users for their judgement.

After the judgements were complete, in attempting to characterise how acceptable the proposed approaches were, users were also questioned on the usefulness of the suggested query texts on semi-automatic runs. They were also questioned on the general effectiveness and their satisfaction with the augmentation process overall.

## 5. Results from Event Augmentation

We now evaluate the results of each of the three phases of our event augmentation system as illustrated in Figure 4, namely:

- Which source(s) of geo-tagged photos should we retrieve from which to extract user tags?
- How effective is the automatically constructed text query from those tags?
- Do users prefer a fully automated augmentation process, or one where a text query is suggested but given the option to amend that query?

### 5.1. Which Photos to Retrieve from the Same Location?

Given that the user will have the time and GPS information of their lifelog event automatically recorded, we retrieve relevant geotagged images from Flickr based on a location search. In describing our approach in Section 3.1., we posed the question as to whether it is better to retrieve the tags from the top 100 (*T100*) or the top 25 (*T25*) most relevant (in terms of location) geotagged images, or the top 25 geotagged images that are visually similar to the keyframe image from the lifelog event (*M25*).

**Figure 10.**
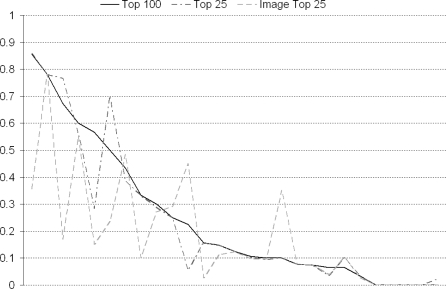
Selection of seed geotagged photos to extract tags—x axis is ordered from most to least successful query topic.

In practice we found that none of these approaches performed consistently better than any other across different events, although selecting the top 100 geotagged images provided marginally superior results (overall precision of 0.308 *vs.* 190 for top 25 geotagged *vs.* 0.276 for top 25 visually similar). 34 of all the tagged events had their results generated automatically, and here it was possible to compare the performance of these 3 approaches (in terms of the number of relevant results they lead to) as displayed in Figure 10. Again using the tags from the top 100 geotagged images performs best (0.260 precision) (As no suggested text was presented to the user in automatic runs, users were presented with results from the *T100, T25, & M25* approaches, thus allowing us to compare the retrieval results of these approaches on a like-for-like basis). Selecting the top 25 most visually similar performs worst (0.217), and to compound matters there is a large processing overload associated with this approach by having to extract the MPEG-7 descriptors of the top 100 images returned by Flickr and Panoramio for each event (in practice we have found this takes 10 × the processing time).

We investigated whether the number of (seed) geotagged images initially retrieved had any impact on the final system performance and in practice we found no such relationship/correlation existed (correlation of −0.09 between number of seed geotagged images and precision of retrieved results).

**Figure 11.**
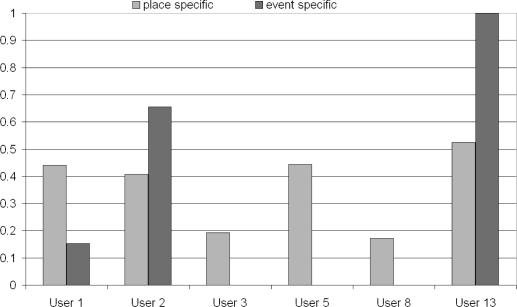
“Place Specific” *vs.* “Event Specific” results by user.

We discussed earlier that lifelog events, which we may like to augment, can be placed into two broad categories, *i.e.*, whether the lifelog event in question is of a specific place (“place-specific”), or an event or happening such as a big sporting event or rock concert (“event-specific”) at a place. So the “place-specific” category can include images taken at any time, whereas the “event-specific” category can only include images taken at a certain time period. Overall the “place-specific” event augmentation performed better than the “event-specific”, with an average precision score of 0.305 *vs.* 0.186. Also out of the 6 users who had both “place-” and “event-specific” events, the “place-specific” results were better for 4 of them (see Figure 11). The “place-specific” also performs better on both the automatic (0.249 *vs.* 0.123) and semi-automatic systems (0.448 *vs.* 0.378). Comments made on this aspect of the augmentation process in our post evaluation questionnaire were concentrated on criticising the “event-specific” results, with one user (User 5 in Figure 11) commenting that *“. . . for location-specific events, most of them were good. For event-specific events it was all incorrect . . . ”*. No comments were made when the event-specific system worked better, perhaps indicating an expectation that it should just work anyway.

Given that it is possible to automatically search for geotagged images, we posed the question as to how many images should be retrieved so as to construct a new text query subsequently. We found that taking the 100 most spatially relevant images performs best.

### 5.2. Benefits Offered in Using Relevance Feedback to Construct Text Query

After automatically retrieving geotagged images, we proposed earlier that intelligently constructing a new text query from the tags associated with those retrieved images could be beneficial, and provide even more relevant images/videos. This is due to the fact that while there are now tens of millions of geotagged images available, it is still a small percentage of the *total* amount of potentially relevant images/video. Thus by constructing a text query, it is then possible to access any of those other relevant images/videos.

It is indeed the case that constructing a new text query is beneficial, as 55.15% of the total number of relevant images/videos across all users (on the automatic runs) came from the sources of information that depend on the automatically constructed query. This immediately offered users an additional 123% images.

Given that users were given the opportunity to amend the text queries on the semi-automatic system runs we can use another method to evaluate how closely the suggested text matches the user query. Heavy editing would negate the advantages of automatically suggesting keywords so to do this we calculate the number of overlapping words between the suggested text and the actual query input by the user (taken as the gold standard). This enables us to calculate precision (the total number of suggested words divided by the number of overlapping words), recall (the total number of actual words input by the user divided by the number of overlapping words), and the F1-Measure $\frac{2*\mathrm{precision}*\mathrm{recall}}{\mathrm{precision}+\mathrm{recall}}$.

**Figure 12.**
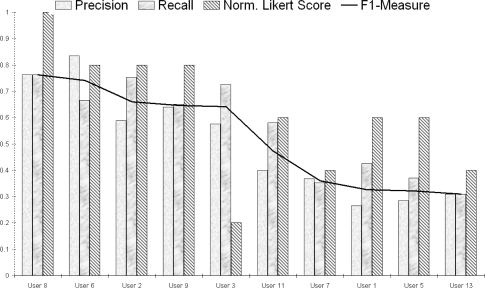
Effectiveness of tag suggestion for users on completely automated runs.

Again using the top 100 geotagged images as a source of tags works best with a precision of 0.489, a recall of 0.582, and an F1-Measure of 0.532, indicating that the suggested tags were helpful to users. However given that each query is different, and some are much more difficult than others, there is naturally a large variation of scores between users on how accurate the suggested text was (ranging from F1-Measures of 0.76 to 0.31) as illustrated in Figure 12. It is also interesting to note the trend between the F1-Measure (thick continuous line) and the normalised user Likert rating for the tag suggestion usefulness in a post-evaluation questionnaire (diagonally filled column). Broadly speaking these two data series mirror each other (with a correlation of 0.55), thus indicating that users naturally are happier when more accurate query text is suggested. User number 3 was the only person to buck this general trend, feeling that (for his queries) the suggested text was not sufficiently specific, *“. . . You really need local knowledge of the area to form the query correctly . . . ”*.

Indeed, in a post-evaluation questionnaire there were some negative comments in which the most common complaint was that the text suggestions were not “specific” enough, e.g., provides the city/street name, but not the name of the particular building that the user was interested in. However, in total, 7 out of the 10 users found the tag suggestions helpful in many instances (User number 4 from Table 2 only had 1 query, which was determined to be a completely automated run based on our Latin Squares setup. Therefore only 10 users instead of all 11 could be evaluated in this section). The median Likert score of all 10 users was 3/5.

### 5.3. Augmented Image/Video Search Results

Augmented content is available from a number of different data sources, and we now comment on those sources that consistently provide a greater concentration of relevant material.

As previously mentioned, due to the relatively small percentage of publicly available geotagged content, it might be useful to construct a new text query based on tags associated with retrieved images, so as to search for *many* more potentially relevant images/videos to a user’s lifelog events. Having automatically constructed a new text query on which to search for other sources of information (e.g., Yahoo!, YouTube, MSN, *etc*.), we proposed two systems to the user: (1) All images are automatically returned to the users, and (2) The suggested text is shown to the user (who can edit it) before retrieving potentially relevant results.

**Figure 13.**
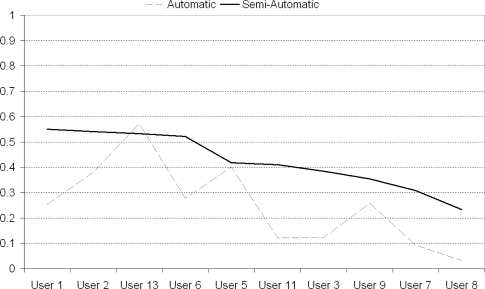
Effectiveness of (semi-) automated runs on each user.

The semi-automatic system’s retrieval results were better for 9 out of the 10 people who used both systems as illustrated in Figure 13. In fact the overall precision of the semi-automatic system was almost twice that of the automatic system (0.441 *vs.* 0.235), thus indicating that user feedback and involvement is vital in locating relevant images to automatically augment lifelog events. In addition to the superior retrieval performance, 9 of those 10 users also simply preferred using the semi-automatic system as it provided them with more security, with one user eloquently summing up the feelings of others by stating that *“. . . I missed the textbox when it was removed . . . ”*.

**Figure 14.**
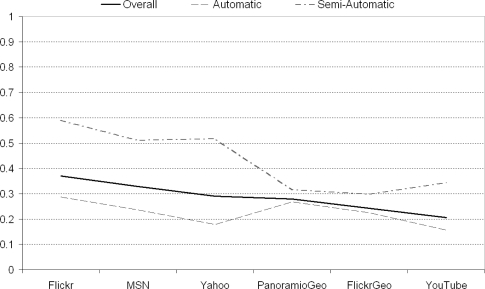
Effectiveness of various sources of augmented images.

Considering that the top 10 results are returned from 6 sources of information (For geotagged images: Flickr and Panoramio; For text search images/video: Flickr, Yahoo!, MSN, YouTube), we now report those sources that provided the highest concentration of relevant results. Investigating all user judgements on images for event augmentation, searching Flickr by text had the highest average precision score (0.370) followed by MSN (0.329), Yahoo! (0.290), Panoramio geotagged images (0.280), Flickr geotagged images (0.242), and finally YouTube (0.205) as illustrated in Figure 14. Given that users much prefer the semi-automatic system, it is interesting to see which sources of information provided the highest number of relevant results on semi-automatic runs (the medium thickness dot dashed line in Figure 14). Flickr text search again works best but this time with a much higher precision score of 590; Yahoo! is next best (0.516), then MSN (0.510), then YouTube (0.343); and finally the two geotagged sources of Panoramio (0.315) and Flickr (0.299). So what is really interesting to note here is that on semi-automatic augmentation runs, which users prefer anyway, the “text only query” sources clearly perform better!

**Figure 15.**
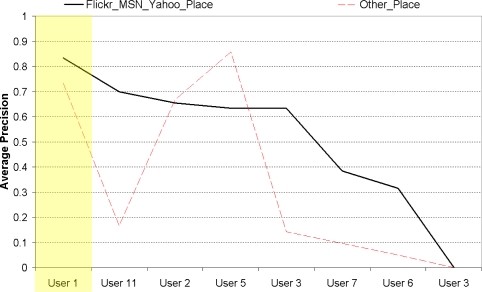
Performance of final recommended system on “place-specific” events.

So far, various facets of our approach have been evaluated in augmenting lifelog events with images/videos from other sources of information. Now we investigate how effective for users the optimal combination of these approaches is. Based on these results, the best overall system would use the top 100 retrieved geotagged images as a source from which to construct a text query. The user would then be given the opportunity to amend the generated text query, and the Flickr (text), MSN, and Yahoo! sources would be used to present candidates for event augmentation to the user. Of the 8 (User 9 did not have the opportunity to make judgements on the optimum system) “place-specific” runs that had these parameter settings in our experiments, a median precision score of 0.633 was recorded, meaning that users found two-thirds of the presented images useful for augmenting their lifelog events, as illustrated by the thick continuous line in Figure 15. It is also possible to include the YouTube, Panoramio and Flickr (geotagged) results, but there will be many noisy/irrelevant results included as these 3 sources had an average precision score of just 0.339 on the 8 aforementioned runs (shown on the “Other Place” dashed line in Figure 15).

There were only 2 “event-specific” runs (with the optimal combination of facets) that could be evaluated in our experimental dataset, therefore it is difficult to draw meaningful conclusions. However on these two runs, only one returned any results at all (with precision of 0.4). This indicates that in future there exists a significant challenge in terms of augmenting “event-specific” lifelog events with relevant content. On inspection the 26 sample queries mentioned in Section 3.2. we discovered that “places of interest” have many more relevant images (e.g., Sugar Loaf mountain, Big Ben, *etc*.) than “events of interest” (e.g., soccer world cup final, AFL grand final, *etc*.). This means that there is a smaller number of potentially relevant results available which makes the challenge more difficult than retrieving relevant “place-specific” queries.

### 5.4. Analysis of Recommended System on Recreational Lifeloggers

As discussed in Section 4. it was evident from Table 2 that user 1 supplied the most data as he was a very enthusiastic lifelogger, wearing a SenseCam all day, every day, over a period of almost 2 years. To consider concerns on whether user 1 has skewed the dataset, it is worthwhile to note that on evidence of excluding the shaded area related to this user in Figure 15 the “place-specific”(user 1 didn’t have any “event-specific” results to judge using the optimum system) results for the other “recreational lifeloggers” remain broadly the same. The recommended system still maintains a median precision score of 0.633, while the average precision score of the alternative/comparative system including the YouTube, Panoramio, and Flickr (geotagged) results drops to 0.282.

## 6. Future Directions

Another possible feature we initially considered including in the augmentation of lifelog events, was that of inferring the user’s current activity at the given time, and using it as an additional term in the textual query.

Indeed using the tri-axis accelerometer onboard the SenseCam, we are able to accurately identify generic activities such as *sitting/standing*, *walking*, and *driving*. One of the strengths of the SenseCam is as a valuable context reinstatement tool (in terms of the images it captures which are powerful cues to recall) and thus we manually labelled different types of activities from one week’s worth of SenseCam accelerometer data. This equated to the manual validation of 132,247 motion readings using 16,181 images. Using a range of classifiers (Logistic Regression [22], Naive Bayes [23], C4.5 decision trees [24], SVM [25], *etc*. (All 4 classifiers had similar performance and eventually we selected the SVM [25] which performed best on our data) we were able to identify *sitting/standing* activity with an accuracy of 75%, *walking* at 77%, and *driving* at 88%.

A drawback of these activities is that they are quite generic and do not supplement the augmentation process. Therefore a more fruitful adventure may well be offered in inferring activities from the content of SenseCam images. A number of colour/texture/edge features [19] or interest point features [26, 27] can be extracted from each and every SenseCam. Given enough samples of a given semantic concept or activity, machine learning techniques can be used on these features to begin to recognise and identify trained activities. Indeed the video (Video is essentially a sequence of images) retrieval community organise a benchmarking exercise on extensive datasets to help accelerate the start of the art in more accurately identifying these semantic concepts or activities [28]. The techniques developed in this field are starting to show quite a bit of promise (e.g., doing processing on the GPU (GPU = Graphics Processing Unit, which can offer significant speed gains) level and using advanced SURF image matching features and representing them as a “bag of words” to allow for efficient processing [29]). The current focus of the community is in scaling up the number of activities detected as simulations indicate this may lead to significant breakthroughs in terms of accuracy levels achieved [30]. A drawback associated with all techniques used thus far though is the burden placed on individuals to manually identify a sufficient volume of examples for the classifiers to learn from, which also makes it non-trivial to adapt these techniques to lifelog data which is quite different by nature.

In prior work we have investigated applying these techniques on the image content captured by SenseCam images. These semantic concepts were trained and evaluated on a very large dataset of 58,785 unique images labelled by 11 annotators [31]. On closer inspection of these results (see Table 3), it is evident that “object/scene” based concepts are more accurate than “activity” based concepts (62% *vs.* 44% average accuracy).

It must be considered that the field of lifelogging offers new challenges (from the video domain) in accurately identifying activities. These challenges include: non-broadcast quality image content (the SenseCam offers low resolution images of 640 × 480 pixels which are further distorted by fisheye lens); and fusion techniques to incorporate the sensory sources of information too. We believe that once the community successfully begins to meet those challenges, there will be much merit in then investigating whether it can be useful to apply these techniques to supplement the process of event augmentation described in this article.

## 7. Conclusions

In sensor research we have long strived to better understand a sensor’s values through augmenting it with other complimentary sensed data. Such approaches help us better understand given sensed events. Indeed as sensing technologies become more ubiquitous and wearable a new trend of lifelogging and passive image capture is starting to take place and early clinical studies have shown much promise in aiding human memory. To help further understand lifelogged events we have investigated techniques to supplement them with augmented material, which presents a unique challenge as motivated in the introduction section.

We have presented a general model for going from a marker—a (latitude, longitude) pair or (latitude, longitude, time) tuple—to a set of relevant augmented “Web 2.0” content items. There is nothing to prevent our approach being used by images taken by cell phones or digital cameras with related location data. In this article we have focused on a novel form of automatically sensed information, and we investigate how our augmentation system helps provide additional information to the benefit of our users.

In our experiments, a total of 4,963 judgements were made on augmented images/videos for 67 user-selected lifelog events taken from almost 2 million SenseCam images, and in conclusion our users had a pleasurable experience in viewing the augmented material, especially when they were allowed to refine the text queries automatically proposed by the system. There are research challenges involved in further improving the quality of the lifelog augmentation process, especially with regard to “event-specific” lifelog events, e.g., football matches, rock concerts, *etc*. Other research challenges include investigations into selecting initial seed images based on adaptive radii, more sophisticated tag selection techniques, and also considering how interface design and varying methods of visualisation affect users’ acceptance of augmented data.

We are now entering an era whereby we as individuals expend little effort in capturing sensory data and images and making them available, whether it be passively captured lifelog data or a smaller number of manually captured photos which are uploaded to a media sharing website. Aggregating these small contributions over an enormous scale of users we have automatically enriched the experience of individuals reviewing their lifelogged trips or events by providing them with a large number of additionally relevant items of information mined from millions of other individuals.

## Figures and Tables

**Table 1.** Sample tags associated with geotagged images returned for “Sentosa Island” query event.

Tag	Num Users	Tag	Num Users
Sentosa	27	from	3
Island	10	of	3
Merlion	10	Island	3
Beach	5	Garden	2
at	4	flowers	2
geotagged	4	Dragon	2
The	3	Butterfly	2

**Table 2.** Summary of data collected by our users.

User	Days Worn	Num Images	Num Events	Place Tagged	Time Tagged
1	614	1,686,424	19,995	5	4
2	60	92,837	1,182	8	2
3	21	44,173	443	9	1
4	3	3,437	39	0	1
5	23	40,715	505	5	1
6	24	38,106	465	5	0
7	8	18,485	169	7	0
8	6	8,296	57	3	1
9	1	2,046	20	6	0
10	1	667	8	6	0
11	2	2,584	28	2	1

**Total**	**763**	**1,937,770**	**22,911**	**56**	**11**

**Table 3.** Accuracy of activity recognition on lifelog data [31] (sorted by “Type” then by “Accuracy”).

Type	Concept	Num Training Samples	Concept Precision Accuracy
activity	shopping	228	74%
activity	reading	927	58%
activity	people	6,633	45%
activity	eating	2,143	40%
activity	holdingPhone	233	38%
activity	holdingCup	418	34%
activity	meeting	2,396	34%
activity	presentation	352	28%
object/scene	indoor	19,287	81%
object/scene	sky	1,513	78%
object/scene	screen	10,601	78%
object/scene	steeringWheel	208	72%
object/scene	office	7,783	71%
object/scene	door	579	68%
object/scene	hands	10,649	67%
object/scene	veg	778	64%
object/scene	tree	957	63%
object/scene	outdoor	3,292	62%
object/scene	grass	444	61%
object/scene	face	3,018	60%
object/scene	insideVehicle	2,226	60%
object/scene	buildings	1,853	58%
object/scene	toilet	174	57%
object/scene	stairs	87	48%
object/scene	road	1,008	47%
object/scene	vehiclesExternal	480	46%
object/scene	viewHorizon	3	23%
